# Oxytocin-based therapies for treatment of Prader-Willi and Schaaf-Yang syndromes: evidence, disappointments, and future research strategies

**DOI:** 10.1038/s41398-022-02054-1

**Published:** 2022-08-08

**Authors:** Ferdinand Althammer, Francoise Muscatelli, Valery Grinevich, Christian P. Schaaf

**Affiliations:** 1 Institute of Human Genetics, University Hospital Heidelberg, Heidelberg, Germany; 2 Aix-Marseille Université, Mediterranean Institute of Neurobiology (INMED), Parc Scientifique de Luminy, Marseille, France; 3 Department of Neuropeptide Research for Psychiatry, Central Institute of Mental Health, University of Heidelberg, Mannheim, Germany; 4 Center for Neuroinflammation and Cardiometabolic Diseases, Georgia State University, Atlanta, USA

**Keywords:** Medical genetics, Molecular neuroscience, Autism spectrum disorders

## Abstract

The prosocial neuropeptide oxytocin is being developed as a potential treatment for various neuropsychiatric disorders including autism spectrum disorder (ASD). Early studies using intranasal oxytocin in patients with ASD yielded encouraging results and for some time, scientists and affected families placed high hopes on the use of intranasal oxytocin for behavioral therapy in ASD. However, a recent Phase III trial obtained negative results using intranasal oxytocin for the treatment of behavioral symptoms in children with ASD. Given the frequently observed autism-like behavioral phenotypes in Prader-Willi and Schaaf-Yang syndromes, it is unclear whether oxytocin treatment represents a viable option to treat behavioral symptoms in these diseases. Here we review the latest findings on intranasal OT treatment, Prader-Willi and Schaaf-Yang syndromes, and propose novel research strategies for tailored oxytocin-based therapies for affected individuals. Finally, we propose the critical period theory, which could explain why oxytocin-based treatment seems to be most efficient in infants, but not adolescents.

## The role of oxytocin in neuropsychiatric disorders: one treatment for all?

The hypothalamic neuropeptide oxytocin (OT) is well-conserved across the animal kingdom and modulates a variety of vital physiological processes, as well as social behavior [1]. OT is synthesized in the hypothalamus and reaches target regions that express OT receptors (OTRs) and, to some extent vasopressin receptors V1AR [2], both through diffusion and local release of the peptide [3, 4]. OT neurons form long-range projections and innervate various brain areas [5], albeit target regions, OTR expression levels, and affected behaviors greatly vary between mammalian species [6, 7]. A plethora of studies reported prosocial effects of OT in various species and different behavioral paradigms [8–12] and thus, it was speculated that administration of OT could prove beneficial in several neuropsychiatric disorders, in which the OT system was compromised. One of such disorders is autism spectrum disorder (ASD), for which a dysfunctional OT system was considered one of the main underlying causes for quite some time [13, 14]. OT administration to rescue symptoms and behavioral abnormalities were tested in different genetic and pharmacologically-induced mouse models for autism and yielded encouraging results. OT improved behavioral and electrophysiological deficits [15] and reversed abnormal neuronal morphology [16] in the Shank3-deficient mouse, ameliorated autistic-like behavior in a valproic-acid-induced autism mouse model [17], had significant prosocial effects in both BALB/cByJ and C58/J models [18], reversed social deficits in C58/J and Grin1ko mice [19] and restored social behavior in *Magel2*^*tm1.1Mus*^-deficient mice [20, 21].

In humans, OT treatment has been suggested as a therapeutic approach for treating anxiety [22, 23], autism [22, 24–29], schizophrenia [22, 27], depression [22, 30] or PTSD [31]. Ooi and colleagues performed a thorough meta-analysis and reviewed existing publications and given the mixed findings from clinical trials, they came to the conclusion that the effectiveness of OT treatment for ASD should be considered tentative [32]. Thus, the question arises whether one type of treatment for all the different conditions is a sound approach, especially considering that genotype-phenotype distinctions may have a huge impact on treatment efficacy and that the underlying cause resulting in the same clinical diagnosis might be entirely different between individuals. In fact, using similar intranasal OT protocols to treat complex neuropsychiatric illnesses such as autism or schizophrenia highlights a mixture of unwarranted optimism and lack of understanding about how the OT system operates. Flooding the brain with massive amounts of OT (up to 20 times the pituitary content, although <0.05% is expected to cross the blood-brain barrier [33] exposes one of the fundamental dichotomies in translational OT research: “The way we believe OT acts in the brain vs. how it really works”. Research in the last few years revealed that many of the seemingly well-established dogmas of OTergic signaling turned out to be wrong or at least drastically oversimplified. Recent studies showed that the traditional views [34] about how OT is released within the brain [5, 35, 36], the way OT acts on cells [37–39], the types of cells it acts on [40], how OTergic cells communicate with each other [35, 41–43] or even the assumption about the number of different OT subtypes [41, 44] were only partially correct [45]. If and how OT therapy is the most effective depends both on the underlying condition and the context of the therapeutic intervention [46]. Thus, results from studies need to be carefully interpreted and put into context considering various factors such as genetic background, sex, age and context, before reaching universal conclusions on the efficacy of OT treatment for specific neuropsychiatric disorders.

## Prader-Willi and Schaaf-Yang syndromes and the role of oxytocinergic signaling

Prader-Willi syndrome (PWS) is characterized by infantile hypotonia, weight gain, and overeating during childhood, as well as developmental delay and intellectual disability [47]. Schaaf-Yang syndrome (SYS) has several overlapping features with PWS and was thus initially considered to be a PWS-like syndrome [48]. While both diseases share features such as hypotonia, feeding difficulties during infancy, and cognitive impairments, there are also clinical differences between the two conditions. The diagnosis of ASD is much more commonly established in SYS patients than PWS patients. In addition, SYS patients display unique features such as joint contractures. On the other hand, PWS core symptoms such as hyperphagia and obesity are much less prevalent in SYS.

**Fig. 1:**
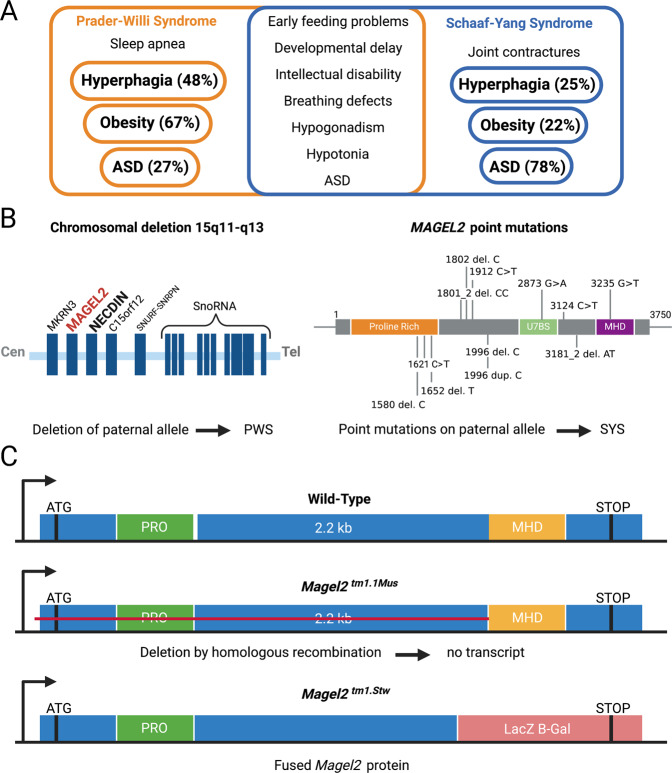
Genetic causes and transgenic mouse models of PWS and SYS. **A** Overview of common and distinct symptoms in PWS and SYS patients, as per [76]. **B** Genetic causes for PWS and SYS. Deletion of *MAGEL2* on the paternal allele causes PWS, while various point mutations within the *MAGEL2* gene are the cause of SYS. **C** Differences in the two transgenic Magel2 KO mouse models. In the *Magel2*^*tm1.1Mus*^ mouse, no transcript is produced due to the deletion of the gene by homologous recombination. In the *Magel2*^*tm1.Stw*^ mouse, insertion of the LacZ B-Gal gene results in the expression of a fused *Magel2* protein.

In individuals with PWS expression of several contiguous genes is disrupted and lack of the paternal copy of the protein-coding gene *MAGEL2* is believed to be one of the main causes underlying the disease. However, the lack of others genes within the chromosomal 15q11.2-q13 region, such as *SNORD116*, are also considered to contribute to the phenotype of PWS [49, 50]. On the other hand, pathogenic/truncating variants of *MAGEL2* represent the cause of SYS [51]. In order to study gene-related phenotype, two *Magel2* knockout (KO) mouse lines have been generated. *Magel2* KO mice display unique behavioral patterns, including reduced levels of anxiety, lack of social discrimination (*Magel2*^*tm1Stw*^) [52, 53], as well as altered exploration behavior and social interaction (Magel2^tm1.1Mus^) [21, 54]. Intriguingly, the suckling deficit and nearly all the deficits in social behavior and learning abilities in Magel2^tm1.1Mus^ mice could be rescued by daily administration of OT during the first week of life only [20, 21]. It is important to note that the two *Magel2* KO mouse lines are two different genetic constructs and display different behavioral phenotypes. In the *Magel2*^*tm1.1Mus*^ mouse, *Magel2* gene is partly deleted and no transcript is produced Magel2^tm1.1Mus^ mice show deficits in suckling at birth [54], atypical response to sensory stimuli [55], and alterations in social behavior and cognition in infancy and adulthood [20, 21]. On the other hand, generation of Magel2^tm1Stw^ mice was achieved by replacing the Magel2 gene by a fused gene that encodes the N terminal part of the Magel2 gene fused with Beta-Gal gene, resulting in a Magel2/B-Gal fused protein. Magel2^tm1Stw^ displays reduced signs of anxiety, lack a preference for social novelty, and impaired learning ability [56]. Distinct and overlapping symptoms of PWS and SYS in humans, genetic causes [57, 58], and the two different Magel2 KO mice are highlighted in Fig. 1.

Analysis of OT neurons in Magel2^tm1Stw^ mice revealed an overall suppressed activity of OT neurons resulting from an imbalance synaptic input onto OT neurons, characterized by reduced excitatory and increased inhibitory postsynaptic currents [59]. Furthermore, OT administration in Magel2^tm1.1Mus^ pups rescued several changes related to neurite outgrowth, including altered synaptic vesicle proteins and cell adhesion molecules [60]. Most importantly, however, based on the preclinical studies performed on Magel2^tm1.1Mus^ [54], one study investigated the effect of intranasal OT administration in infants with PWS and reported normalization of suckling in 88% of infants [61]. In addition, the authors reported a list of further improvements, including social withdrawal and altered mother-infant interactions. This study, which is currently being repeated as a Phase III trial (NCT03649477), provides crucial evidence that a dysfunctional OT system underlies many of the observed symptoms in children with PWS, and that OT administration in neonates/infants is sufficient to restore many of the observed pathologies.

Over the past decade, research has shown that in addition to its well-studied pro-social effects, OT acts as a neuromodulator in various brain regions [37]. Thus, OT not only enables intra-neuronal communication, but also has far-reaching consequences on entire neuronal circuits. It modulates important brain region-specific functions including memory consolidation [39], retrieval or sensory development [62], social interaction [43], and anxiety [42]. Given the manifold effects that physiological and psychological changes such as lactation [63, 64], anxiety [23, 42, 65, 66], maternal separation [67–69] or social isolation [70, 71] have on the number of OT cells, projections and/or OTR expression levels, one could speculate that some of the neonatal clinical manifestations of PWS and SYS also are linked to a dysfunctional OT system, which subsequently results in some of the behavioral abnormalities observed in PWS and SYS patients. It seems plausible that OT cells innervate various socially-relevant brain regions less frequently, have impaired OT release from long-range axonal terminals and/or that a disease-driven downregulation of OTRs in different brain regions takes place. In fact, the findings that administration of OT during infancy rescued social recognition and learning disabilities until adulthood [20, 21] and restored electrophysiological properties, normal levels of OTRs [20] and altered synaptic vesicle proteins and cell adhesion molecules in Magel2 KO mice [60] support this theory. However, to this date, no detailed study investigating the physiological, molecular or morphological changes in OT neurons in PWS/SYS has been conducted. Taken together there is a causal link between a dysfunctional OTergic system and the behavioral abnormalities observed in Magel2 KO mice [20]. However, more studies are needed to precisely pinpoint the brain areas, cell types and parts of the OTergic pathway (innervation, morphology, release, OTR binding and intracellular cascades) altered in murine models of PWS and SYS.

## Differences in the phenotypic spectrum of PWS and SYS patients

**Fig. 2:**
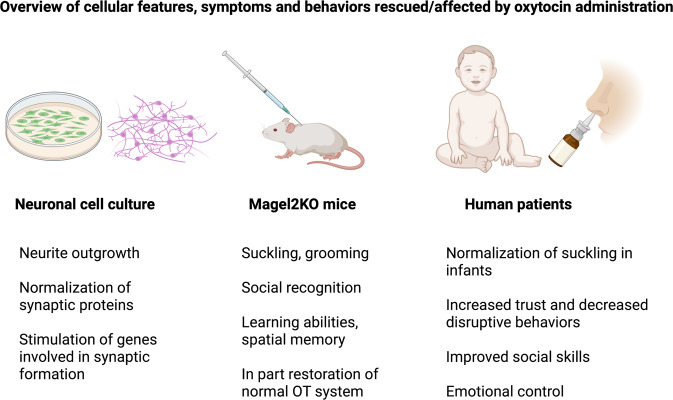
Effects of oxytocin treatment on symptoms in PWS and SYS models. List of features and symptoms that were rescued by OT administration in neuronal cell culture, mouse models, and PWS patients.

While the paternal copy of the chromosomal 15q12-q13 region, including *MAGEL2*, is completely absent in the vast majority of cases of PWS (with the rare exception of imprinting defects <2% of cases), pathogenic/truncating mutations of *MAGEL2* cause SYS. Recent studies showed that ASD-related symptoms occur more frequently in individuals with SYS (78% vs. 28%), albeit some PWS patients also display (ASD)-like features [48]. Infants with PWS often have a poor muscle tone (hypotonia), distinct facial features like almond-shaped eyes, show a generally poor responsiveness and weak cry, and frequently have underdeveloped genitalia [72–75]. In SYS patients, gastrointestinal/feeding difficulties are particularly pronounced in infancy and adulthood, but unlike in PWS, rarely transition to hyperphagia and obesity in adulthood [51, 58, 76, 77]. In most individuals with SYS, obesity and hyperphagia-related symptoms are either mildly expressed or entirely absent in children [48], but there is recent evidence that the prevalence of obesity increases among adult individuals with SYS [78]. 50–60% of individuals with SYS have short stature and treatment with growth hormone proved to be effective [77]. Unlike in PWS, facial dysmorphisms are mostly nonspecific such as a pointed, prominent chin, frontal bossing, busy eyebrows or short noses [48, 51]. How and why such differences in the reported symptoms between individuals SYS and PWS occur is currently unclear. One possibility is a potential neomorphic effect of the truncated MAGEL2 protein in SYS, which could drastically vary depending on the tissue or cell types in which it is expressed. In addition, leaky expression of *MAGEL2* originating from the maternal allele in PWS has been speculated and could explain differences in phenotype severity [79]. Finally, compensatory effects of other protein-coding genes in PWS such as Necdin, which is located next to *MAGEL2* on 15q11-q13, or snoRNAs are not well studied. If and how these subtle genetic differences affect OT morphology, projections and function remain elusive. OT signaling is crucially involved in motor and sensory development in all mammalian species [62] and developmental impairments caused either by the altered release of OT, reduction of OTergic fibers or diminished OTR expression would have detrimental consequences for the organism. Thus, detailed morphological and physiological studies assessing OTergic differences between murine models of PWS and SYS are necessary, and need to be verified using patient-derived iPSC cultures and, ideally, through post-mortem analysis of brains from PWS/SYS patients. These studies will provide new insights about structural and functional differences of OT neurons in both diseases and will help to develop OT-based therapeutic interventions that are tailored towards the individual needs of patients. An overview of cellular features, symptoms, and behaviors that were rescued by administration of OT either in cell culture, murine models, or human patients can be found in Fig. 2.

## Intranasal oxytocin treatment for ASD spectrum disorders: a sobering phase III trial and its relevance for the treatment of Prader-Willi and Schaaf-Yang syndromes

Ever since the famous landmark studies of intranasal OT administration by Kosfeld and colleagues [80] and Kirsch and colleagues [81], various forms of OT treatment (intranasally, infusion or endogenously-evoked release) have been applied to various behavioral paradigms, as well as many psychiatric illnesses and diseases, both in rodents, monkeys, and humans [24, 82–96]. It is important to note, however, that some skepticism about the efficacy of intranasal OT delivery remains [33, 97]. While many of the initial papers on intranasal OT treatment for ASD reported very promising results [87, 98–101], follow-up studies or trials by other groups contradicted the initial findings [24, 26, 102–104]. Recently, one of the most-awaited Phase III trials, which assessed the effects of intranasal OT for the treatment of social impairments in children and adolescents with ASD, reported no significant differences between OT and placebo [105]. These findings are a huge disappointment for affected individuals and families, and a setback for clinical OT researchers across the world. However, Ford and Young argue that these results are not surprising [46], as OT facilitates social learning, but does not directly cause prosocial behavior. The authors further argue that context is key for OT-based therapeutic approaches and that chronic administration might not be the best option to treat psychiatric illnesses that are accompanied by anti-social behavioral symptoms [46].

PWS and SYS share many ASD-related behavioral abnormalities, as well as low emotional recognition and lower verbal and full-scale IQ [48, 106, 107], albeit typical ASD-related cognitive impairments seem to appear much less frequently in patients with PWS [48]. Thus, the negative results from the recent ASD Phase III trial give rise to the question of whether intranasal OT treatment may or may not ameliorate behavioral symptoms in PWS and SYS. One study assessed the efficacy of intranasal OT in children with PWS and reported trends for improvement in several behavioral parameters, including irritability, lethargy, stereotype, and hyperactivity, as well as a significant improvement of overall SRS score on day 6 after the treatment [108]. Another study found no effects of OT on social behavior and hyperphagia in the total group, but the authors reported improvements for anger, sadness, conflicts, social- and food-related behavior in children under 11 years [109], thus highlighting the importance of proper subgroup analysis [110]. On the other hand, Einfeld and colleagues found no differences between OT and placebo with patients aged 12–30 years [111], raising questions about the efficacy of intranasal OT treatment for PWS symptoms in adolescents and adults. Finally, Tauber and colleagues reported that OT might help increasing trust in others and decreasing disruptive behaviors in PWS patients [112], as well as significant improvements of social skills and feeding in infants with PWS [61]. The latter study is currently being repeated as a Phase III trial (NCT03649477). While most of these results are very encouraging, the central question remains: What variables determine if, when, and how OT improves PWS-related symptoms? In a comprehensive review, Rice and colleagues conclude that given the lack of convincing evidence, currently no conclusive statement about the efficacy of intranasal OT as a treatment for PWS can be made [113]. A summary of all studies using intranasal OT (or the OTR agonist carbetocin) can be found in Table 1. Taken together, it seems that intranasal OT can be beneficial for PWS patients in certain situations and certain ages, with differences in dose and treatment duration and potentially with a critical period, in which the underlying circuitry is sensitive to the artificially-delivered OT and can still compensate for loss or dysfunction of the OTergic system, which ultimately leads to a positive behavioral change. In addition, it seems plausible that the context in which OT is administered is pivotal, similar to what Young and Ford suggested for intranasal OT accompanied by behavioral therapy for the treatment of ASD-related symptoms [46]. Finally, new studies and meta-analyses are needed that assess the genetic background, intellectual abilities and behavioral phenotype of patients with PWS and SYS to better to correlate individual patient backgrounds with the respective therapeutic outcome. This will help to predict who might benefit the most from therapies involving the application of intranasal OT.

**Table 1:** Overview and outcomes of studies using intranasal OT or carbetocin for the treatment of patients with PWS.

Type of treatment	Mode of treatment	Age, sample size	Sex	Outcome	Reference
*Intranasal OT*	*16 IU per day, 8 weeks total*	*5–18* *y, n* *=* *11 vs. n* *=* *10*	*m/f*	*No improvement of hyperphagia or repetitive symptoms*	[150]
*Intranasal OT*	*18/24 IU, later 32/40 IU, twice daily, 8 weeks total*	*12–30* *y, n* *=* *22*	*m/f*	*No significant improvements, high OT increased temper outbursts*	[111]
**Intranasal OT**	**16–40 IU, twice daily, 3 months total**	3–11 **y,** ***n*** = 13 per group	**m/f**	**No effects in the total group, positive effect on social and eating behaviors in boys**	[151]
**Intranasal OT**	**24 IU, twice daily, 4 weeks total**	6–14 **y,** ***n*** = **14 vs.** ***n*** = 11	**m/f**	**No effects of OT on hyperphagia or social behavior in the total group, improvement in children younger than 11 years**	[109]
***Intranasal Carbetocin***	***150*** ***µl per dose, 3 times per day, 14 days total***	***10–18*** ***y, n*** ***=*** ***20 vs. n*** ***=*** ***17 per group***	***m/f***	***Improvement of hyperphagia and obsessive-compulsive behavior***	[152]
***Intranasal Carbetocin***	***150*** ***µl per dose, 3 times per day, 14 days total***	***10–18*** ***y, n*** ***=*** ***20 vs. n*** ***=*** ***17 per group***	***m/f***	***Improvement of hyperphagia and obsessive-compulsive behavior***	[152]
***Intranasal OT***	***4IU, every other day, daily or twice daily***	***1–6 months, n*** ***=*** ***18***	***m/f***	***Suckling behavior normalized in 88% of infants, improvements in social withdrawal and mother-infant interactions***	[61]
***Intranasal OT***	***24 IU, single dose***	***18–43*** ***y, n*** ***=*** ***12 per group***	***m/f***	***Increased trust, decreased sadness, and less disruptive behaviors in OT group***	[112]

Studies reporting negative results are shown as italics, studies with mixed results as bold and studies with positive results as bold italics.

## Functional magnetic resonance imaging of patients with PWS to study hyperphagia

One of the most prominent features of adult PWS patients is severe obesity, primarily thought to be caused by hyperphagia. In order to better understand how changes in the underlying neural substrates translate into altered behavior in PWS patients, several studies assessing brain function via fMRI have been conducted. The main goal of these studies was to investigate how potential changes in neural activity and pathological satiety responses translate into hyperphagia in PWS patients. One of the first fMRI studies on PWS patients assessed the satiety response via measurement of brain activation following ingestion of glucose [114]. The authors showed a delayed response to glucose in PWS patients, with a mean latency of 24 min, compared to a previous study that showed a mean response of 15 min for obese and 10 min for lean volunteers. This was the first study showing that the brains of individuals with PWS display a delayed satiety effect in various brain regions associated with food intake and reward, including the insula, ventromedial prefrontal cortex, and nucleus accumbens. In another study, Nieuwpoort and colleagues investigated differences in food-related brain activation and used healthy brothers and sisters of PWS patients as controls [115]. The authors reported a significantly stronger activation of the left insula and the bilateral fusiform gyrus in control subjects during food-related tasks. Interestingly, the study also showed negative correlations for glucose intake and right amygdala activation, as well as a positive correlation for leptin and right anterior hippocampus/amygdala activation. The authors concluded that PWS individuals displayed aberrant food-related brain activation and dysfunction of neural food-reward circuitry, which could be linked to hyperphagia and obesity observed in PWS patients [115]. Hinton and colleagues assessed neural representations of hunger during energy-controlled meals and correlated these with subjective ratings of hunger, fullness, and desire to eat while also measuring blood levels of glucose, insulin, leptin, ghrelin, and PYY [116]. While they found no differences in brain activity between control and PWS patients after an overnight fast, they observed abnormal patterns of satiety responses after food intake in PWS patients. The authors concluded that the most likely explanation for their finding is a disease-induced insensitivity of satiety-associated brain regions even to high-energy food intake [116]. Work by Blanco-Hinojo and colleagues showed a lack of response to disgusting food in PWS patients in the hypothalamus and related brain regions. The authors further reported that activation was restricted to the cerebral cortex and almost absent in deeper subcortical structures of PWS patients [117]. Intriguingly, a recent study identified the anterior deep cerebellar nuclei (aDCN) as the only brain structure activated by food intake in humans and the authors found significant differences in its activation during fasting and after food intake between control subjects and patients with PWS [118].

Another study assessed pituitary morphology and hypothalamic connectivity in children with PWS with an average age of 13 years. The authors included children with PWS, caused either by deletion at locus q11-13 (DEL) or maternal uniparental disomy (mUPD), as well as a control group of typically developing children [119]. The authors reported altered resting functional state connectivity between the hypothalamus and the left and right occipital complex in children with PWS. In addition, they found that PWS patients had a 50% smaller pituitary volume, an irregular shape of the pituitary and an elongated pituitary stalk. The authors hypothesized that the absence of connectivity between hypothalamus and lateral occipital complexes in both hemispheres could partially contribute to the preoccupation with food in children with PWS, given the role of these connections in the food and reward system [119]. In addition, a study by Manning and colleagues in young adults with PWS showed several structural abnormalities in gray matter volume and cortical structure [120]. They reported large and widespread clusters of both increased and decreased gray matter volume, while volumetric increases were most likely driven by greater cortical thickness.

Patients with PWS often have difficulties focusing on specific tasks and setting appropriate attentional weight to enable rapid task switching. Woodcock and colleagues assessed the relationship between task switching, temper outbursts, and repetitive questioning in PWS patients and showed a specific cognitive deficit, which was characterized by significantly reduced activation in the posterior parietal and ventromedial prefrontal cortices [121]. Although none of the above-mentioned studies directly assessed the function of OTergic neurons in the brain of PWS patients, several showed altered hypothalamic activity and connectivity with other food-related brain regions [117, 119]. Thus, it is tempting to speculate that the activity of OT neurons located in the hypothalamus, which has been implicated in feeding behavior in various studies in animal models [1, 122–125], could also be altered in PWS patients during food intake. Given the anorexic effect of OT, it seems plausible that diminished OTergic activity could even exacerbate hyperphagia, by further delaying the satiety response. Several studies already addressed changes in neural activity after the application of intranasal OT [81, 126, 127] and even OTergic pathways in humans [128], but so far, no direct recording of OTergic activity in human patients has been achieved. Thus, new imaging techniques are needed to specifically identify, label, and study the activity of OTergic neurons in PWS patients before, during and after food intake in analogy to what has previously been shown in animal studies.

## Dysregulation of intracellular pathways in murine and iPSC models of PWS and SYS

Recent studies showed that *MAGEL2* mutations are linked to aberrant intracellular signaling pathways, including the mTOR autophagy pathway [129], as well as the USP7-TRIM27 complex [130]. The authors showed that MAGEL2-USP7-TRIM27 (or MUST) complex, which facilitates the retromer recycling pathway through ubiquitination and activation of the WASH actin nucleation promoting factor, does not function properly in mouse models of SYS [130]. In another paper, increased mTOR activity and altered downstream autophagy markers PS-6, P62, and P-ULK1 in human fibroblasts of SYS patients were reported [129]. In addition, the authors found abnormal dendritic arborization in iPSC-derived neurons from SYS patients, highlighting the far-reaching detrimental effects of *MAGEL2* mutations. These findings are intriguing for a variety of reasons: (i) The WASH-MUST complex is present in virtually all brain cells, thus potentially explaining why PWS/SYS combine an array of seemingly unrelated symptoms such as impaired suckling reflexes, hypotonia, hyperphagia, hypogonadism, contractures of small finger joints as well as intellectual disabilities and ASD-related behavioral abnormalities [48]. (ii) The identification of this intracellular dysfunction opens up various opportunities for gene therapy directed at restoring the normal function of the WASH-MUST complex. Given the omnipresence of the WASH-MUST complex in human cells, restoring the normal function could have far-reaching beneficial consequences. (iii) The dysfunction of the retromer recycling pathway seems to either directly or indirectly affect neuronal pathfinding and/or microtubule regulation, resulting abnormal dendritic arborization. iPSC-derived neurons from SYS patients displayed fewer and shorter dendrites, which could have a detrimental effect on cognition, information processing, and wiring of the brain, depending on what types of cells in what brain regions are affected. It is important to note that a recent study indicated a novel role of the N terminal domain of the SYS protein MAGEL2 in RNA metabolism [131], which stands in contrast to the involvement of the C terminal domain that interacts with the WASH complex [130]. In addition, a recent study reports decreased secretory granule and neuropeptide production in cellular and murine models of PWS, which may, in part, underlie the reported dysfunction of the OT system in models of PWS [20, 21, 59]. At this point it is not clear if some brain areas are able to better compensate for the loss of dendritic complexity and if some cell types are more susceptible or resilient to the genetic alteration.

## Parvocellular OT neurons, genetic susceptibility, and autism risk genes

OT neurons can generally be characterized into two different groups: magnocellular OT (magnOT) neurons and parvocellular OT (parvOT) neurons. MagnOT neurons are large endocrine cells with a diameter of 20–30 µm and have direct contact to the neurohypophysis in the posterior pituitary, where they release large amounts of the neuropeptide into the blood stream [41]. On the other hand, parvOT neurons are much smaller (6–15 µm), have no direct contact to the blood circulation and project to several extra-hypothalamic and hindbrain regions. These two classes of OT neurons are fundamentally distinct, as they greatly vary in shape and size [35, 41], electrophysiological properties [43, 132, 133], enrichment of genetic markers [134], projection sites [35, 41] and overall functions [35, 41–43]. Over the last few years, it became evident that parvOT neurons are pivotal for the orchestration of hypothalamic OT activity and are paramount to various physiological and behavioral processes including pain processing, social behavior, as well as retrieval and extinction of fear memories. Already in the 1990s [135, 136], Swaab and colleagues showed that this specific subset of OT neurons might play a role in PWS, as the brains of patients with PWS displayed significantly lower numbers of OT neurons in the PVN. Intriguingly, the biggest difference was found in the caudal/posterior PVN, where the majority of parvOT neurons reside. However, no study directly addressed the potential role of parvOT neurons in murine models of PWS and SYS yet. More recently, Lewis and colleagues employed single-cell sequencing of magnOT and parvOT neurons via the retrograde tracer Fluorogold^TM^ (FG). The authors could identify several genes that were exclusively located in each cell type, thus providing further evidence for the differences between the two cell types. Most importantly, the authors also identified parvOT-specific autism risk genes [134]. This is the first account of neuropsychiatric-related genes identified in parvOT neurons and it is thus tempting to speculate that parvOT neurons are particularly susceptible to genetic mutations underlying mental illnesses. Various OTR polymorphisms have been linked to psychiatric diseases such as autism or schizophrenia and especially the polymorphisms rs53576 (G to A) and rs2254298 appear to be the most often occurring OTR-specific mutations, which result in a drastically higher risk for ASD [27]. However, the finding that a specific subset of OT neurons harbors specific ASD-related risk genes and is potentially more susceptible to disease-driven genetic, molecular, or physiological changes, is new. We believe that the findings of this study are highly relevant for research on PWS and SYS and future studies are needed to assess potential dysfunctions of parvOT neurons in the two diseases. Enrichment and overexpression of autism risk genes in parvOT neurons in murine models of PWS/SYS could be assessed via scRNAseq and would provide unprecedented information about genetic alterations of OT neurons. In addition, more comprehensive anatomical studies are needed assessing OT cell numbers, OTergic projections, and OTR levels in murine models and brains from patients with SYS and PWS. Technical advances such as the uDISCO tissue clearing method [137, 138] in combination with the new generation of OT promoter-driven, BBB-penetrating AAVs expressing GFP [139, 140] would be a suitable tool to map and study the entirety of OTergic projections in murine models of PWS and SYS. While the uDISCO approach is applicable for post-mortem assessment of human brains [138], novel techniques allowing the labeling and discrimination of parvOT and magnOT neurons in human brains are desperately needed [41].

## Summary and perspectives

**Fig. 3:**
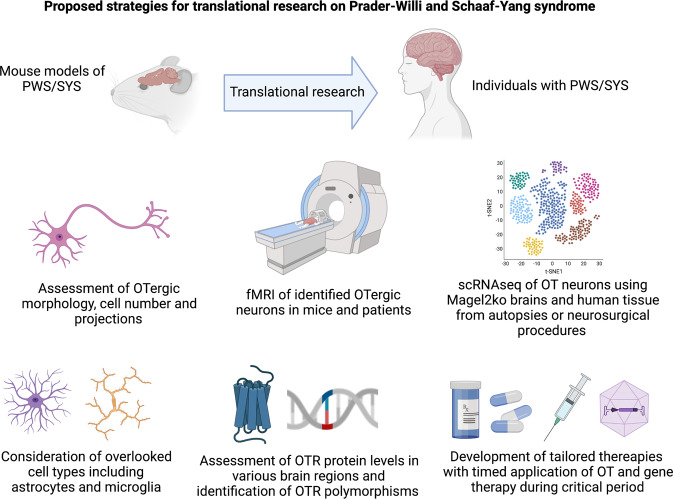
Novel strategies for oxytocin-based translational research in PWS and SYS. Anatomical, functional, molecular, genetic, and pharmacological research directions to study the role of OT in PWS and SYS.

Animal studies performed on Magel2 KO mice clearly showed that OT administration in the first week of life restores early feeding and behavioral and cognitive deficiencies, up to adulthood and corrects the neurobiological alterations [20, 21, 54]. While early studies suggest altered morphology and reduced cellular complexity of OT neurons in Magel2 KO mice and patient-derived neuronal iPSC cultures [129], more studies are needed to assess changes in cellular morphology, projections, and OTRs expression levels in these models. In addition, the role of parvOT neurons in murine models of PWS and SYS needs to be investigated, as various publications highlight a critical role of this subpopulation of OT neurons as master regulators of OTergic activity [35, 41–43] and identified several autism risk genes within this cell population [134]. Post-mortem analysis of brains from individuals with SYS and PWS could be helpful in understanding whether the reduction of OT cells observed in PWS patients stems from magnOT neurons, parvOT neurons or even both. In addition, new OT-directed genetic studies using brain tissue from individuals with SYS and PWS are clearly needed and tissue could be obtained either through autopsies [141–143], epilepsy surgeries [144] or other neurosurgical procedures [143]. Given that OT neurons are located in the hypothalamus, which is a deep structure located in the diencephalon, post-mortem tissue sampling could be a good way to overcome previous challenges associated in obtaining human tissue containing OT neurons. Equally important will be new anatomical and morphological studies that assess changes in projections, differences in morphology of OT neurons, and alterations in OTR expression using brain tissue obtained during autopsies. Other promising approaches to treat PWS and SYS include virus-mediated gene therapy or targeted epigenetic therapy restoring normal histone modification. In the Snrpn/Ube3a (m + /pΔS−U) mouse model of PWS, Kim and colleagues were able to improve the growth and survival of mice using UNC0638, which is a selective inhibitor of euchromatic histone-lysine N-methyltransferase-2 (EHMT-2 or G9a). The drug caused selective reduction of histone methylation without changing DNA methylation, making it a promising candidate for the treatment of PWS patients [145]. In addition, the latest generation of BBB-crossing AAVs equipped with an OT promoter could serve as transport vehicles of genes and proteins for targeted gene therapy [139, 140]. In fact, several studies using gene therapy approaches to treat various aspects of PWS are currently underway and funded by the Foundation for Prader-Willi Research (https://www.fpwr.org/genetic-therapy-for-prader-willi-syndrome#what_is_it). The approaches include obesity-directed treatment via autoregulatory BDNF (Lei Cao, The Ohio State University), epigenome editing (Claudio Mussolino, University of Freiburg) or reactivation of maternal PWS genes (Nahid Iglesias, Duke University). Finally, a recent study identified OTR-expressing astrocytes as a crucial component of an amygdala microcircuit that is involved in emotional processing [40], thus moving glial cells into the limelight of translational research on OT, as glial cells could potentially serve as novel targets for OT-based therapeutic interventions. A series of proposed translational strategies for studying the role of OT neurons in PWS and SYS are shown in Fig. 3.

**Fig. 4:**
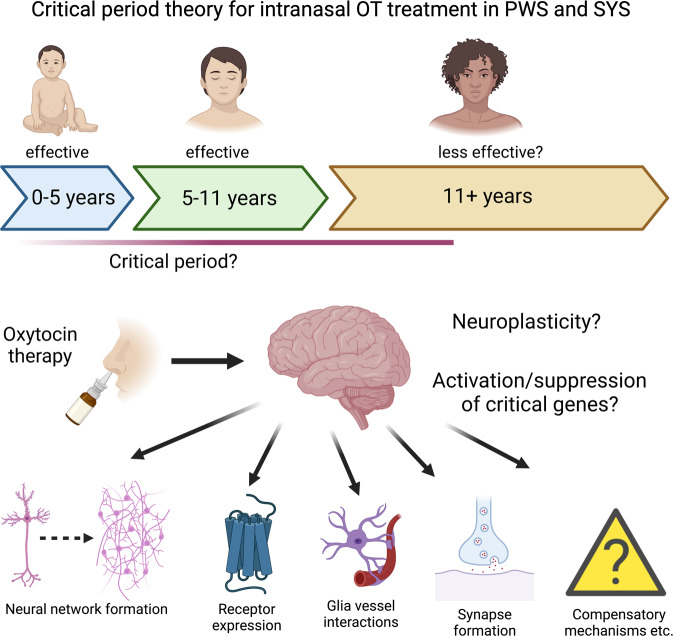
Critical period theory for intranasal OT treatment in PWS and SYS. Intranasal OT treatment for PWS proved to be most effective in children aged 5–11 years. Thus, it is tempting to speculate about the existence of a critical period, in which brains of PWS patients are most receptive for OTergic therapy and the neural circuit can be re-wired and compensate for the dysfunctional *MAGEL2* protein. Reasons for the enhanced efficacy of OTergic treatment could include neuroplasticity, activation or suppression of key genes, as well as the formation of new neural networks, receptor expression, synapse formation, and other factors.

Data on the efficacy of intranasal OT in humans is less clear with studies yielding contradictory [109], promising [61, 108, 112], and negative results [111, 113], thus questioning the efficacy of intranasal OT administration for the treatment of SYS and PWS. Given that both SYS and PWS patients display ASD-related symptoms [48], and the recent negative result of a Phase III trial assessing the efficacy of intranasal OT for the treatment of autism [105], it will be important to also consider the role of context, timing, and behavioral therapy. In fact, the most promising results with intranasal OT were achieved either in infants, toddlers or adolescents [61, 108, 109], suggesting that early treatment might be most likely to succeed. It seems plausible that there is a critical period, during which the OT system can be re-wired and disease-caused alterations can be still compensated for, as is the case with other diseases, disorders or mental illnesses [146–149]. In fact, we hypothesize that the brains of infants and toddlers are more likely to respond to OT treatment due to neuroplasticity and propose the existence of a critical postnatal period (Fig. 4), during which interventions are most likely to succeed. It needs to be stated, however, that at least one study reported a positive effect of OT treatment in patients with PWS [112]. At this point, no definitive conclusions about the efficacy of OT treatment in adults with PWS can be drawn. The sooner a clear PWS/SYS diagnosis is made, the more time and treatment options are available for clinicians. This means that early detection of disease via genetic screening is paramount, both to maximize efficacy with available treatment options and for the development of treatment tailored to the individual needs of patients. Finally, it will be important to perform new meta analyses and treatment efficacy correlations that assess parameters including age, sex, start and type of treatment, and genetic phenotype to find out which subgroups of PWS and SYS patients might profit best from OT-based therapeutic interventions. This will be an important step for the development of tailored approaches addressing the specific needs of affected individuals.
